# Implications of prioritizing HIV cure: new momentum to overcome old challenges in HIV

**DOI:** 10.1186/s12879-016-1445-y

**Published:** 2016-03-03

**Authors:** Joseph D. Tucker, Adam Gilbertson, Ying-Ru Lo, Marco Vitória

**Affiliations:** Institute for Global Health and Infectious Diseases, University of North Carolina at Chapel Hill, Chapel Hill, NC USA; International Diagnostics Centre, Keppel Street, London, WCE1 UK; Center for Bioethics, Department of Social Medicine, University of North Carolina at Chapel Hill, Chapel Hill, NC USA; School of Anthropology and Museum Ethnography, University of Oxford, Oxford, UK; HIV, Hepatitis and Sexually Transmitted Infections Unit, Division of Communicable Diseases, World Health Organization, Regional Office for the Western Pacific, Manila, Philippines; Treatment and Care Unit, HIV/AIDS Department, World Health Organization, Geneva, Switzerland; 2 Lujing Road, Guangzhou, 510095 China

**Keywords:** HIV, Cure, Policy, Social science

## Abstract

**Background:**

Curing HIV is a new strategic priority for several major AIDS organizations. In step with this new priority, HIV cure research and related programs are advancing in low, middle, and high-income country settings. This HIV cure momentum may influence existing HIV programs and research priorities.

**Discussion:**

Despite the early stage of ongoing HIV cure efforts, these changes have directly influenced HIV research funding priorities, pilot programs, and HIV messaging. The building momentum to cure HIV infection may synergize with strategic priorities to better identify adults and infants with very early HIV infection. Although HIV cure represents a new goal, many existing programs and research techniques can be repurposed towards an HIV cure. HIV messages focused on engaging communities towards an HIV cure need to be careful to promote ARV adherence and retention within the HIV continuum of care.

**Summary:**

An increased emphasis within the AIDS field on finding an HIV cure has several important implications. Strengthening connections between HIV cure research and other areas of HIV research may help to catalyze research and facilitate implementation in the future.

## Background

HIV cure research and related programs are accelerating around the world. This new priority developed over the last five years because of an increased understanding of the long-term side effects of lifelong antiretroviral therapy (ART), the public health challenges of current strategies, and basic science advances regarding HIV latency [1]. The goal of curing HIV infection is a strategic priority for the National Institutes of Health National Institute for Allergy and Infectious Diseases [2], the International AIDS Society [1], and other organizations. There are over 100 ongoing HIV cure research studies underway around the world [3].

Although the field of HIV cure is still in its infancy and many decisions about cure efforts or pilot programs have not been centrally organized, there are already important implications of expanding HIV cure momentum [4]. The purpose of this article is to describe these implications and to suggest ways that pilot programs could maximally synergize with existing research and programs. We examine how HIV cure programs, especially research, have started to gradually influence funding priorities, pilot programs for very early HIV detection, and HIV messaging.

## Discussion

The most substantial implication of the increased emphasis of the AIDS field on finding a cure for HIV is changing HIV research priorities. The International AIDS Society HIV cure resources tracking group estimated that 157.9 million USD was invested in HIV cure research in 2014 [5]. These investments from the public sector (139.9 USD) and philanthropies (17.0 million USD) are likely an underestimate because they did not capture growing private sector contributions. A range of public sector funding agencies in high-income countries have prioritized HIV cure research, including the United States National Institutes of Health [2, 6], the Medical Research Council, the French National Agency for AIDS Research, and the Canadian Institutes of Health Research [7]. In addition to high-income country support, there have been special programs to support HIV cure research in China [8], Thailand [9], and Cuba [10]. The National Institutes of Health in the United States has been the single largest funder of HIV cure research to date, accounting for 72 % of the 2014 research funds [5]. The American Foundation for AIDS Research announced a total of 100 million USD in grants to support HIV cure research [11]. The University of North Carolina at Chapel Hill and GlaxoSmithKline recently formed a new public-private partnership to cure HIV, supported by 20 million dollars from GlaxoSmithKline over the course of 4 years [12].

The new emphasis on HIV cure within broader HIV funding priorities raises an important question about the relationship between HIV cure and other HIV research priorities. The International AIDS Society “Towards a Cure” initiative has explicitly stated that the global HIV response should not divert research funding from other areas of HIV research towards cure, instead arguing for greater HIV cure resources [5]. In addition to greater resources for HIV cure research, there is a need for integrative research that crosses disciplinary boundaries [4] and brings together HIV cure and other existing HIV priorities.

Another important policy implication of the HIV cure momentum is enhanced detection of adults and adolescents with acute or very early HIV infection. The first few weeks or months after infection, but prior to seroconversion, constitute the period of acute HIV infection [13]. We define very early HIV infection to include the 6 months following viral acquisition. Adults and adolescents diagnosed and treated during very early HIV infection are critical for HIV cure research [14]. Such individuals are important because they likely have smaller reservoirs [14], decreased viral replication [15] and genetic diversity [16] sustained T-cell and B-cell function [17, 18], better immune restoration potential, lack of extended inflammatory responses and comorbidities associated with chronic HIV infection [19], and no or limited previous exposure to antiretrovirals (ARVs). As a result, individuals with very early HIV infection would likely be easier to cure and recruited for enrollment in HIV cure research studies. Yet the implementation of testing programs to identify very early HIV infections has been challenging [20]. High cost (especially for small laboratories), logistical problems, and the brevity of very early HIV infection further complicate the detection of this phase of HIV infection in many settings. HIV cure strategies for those with very early HIV infection and those with chronic HIV infection will both be important.

New HIV cure momentum also provides an opportunity to synergize and extend existing programs focused on identifying very early HIV infected individuals. Programs seeking to increase or improve diagnosis and treatment during very early infection are driven by both public health imperatives and concerns for long-term patient well-being. Public health programs focused on identifying very early HIV infection have been established because of the increased risk of onward transmission during very early infection [21]. Additionally, the advantages of early diagnosis and suppression of viremia [15, 17–19, 22–24] add impetus to efforts to introduce and retain these patients in care. HIV cure-based scientific interest in early immune response, HIV disease progression, and the establishment and perpetuation of latent HIV reservoirs may add further momentum to these efforts if increased numbers of very early infected and/or ARV suppressed participants are required for research participation. Recently, the Treatment Action Group identified twelve HIV cure research studies around the world already enrolling HIV infected individuals during acute or very early infection [3].

Although the additional momentum created by HIV cure research is unlikely to overcome logistical problems, HIV cure may provide another strong reason to enhance detection of very early HIV infected adults within clinical trial sites. HIV cure related interest in very early infection may help to raise awareness about acute HIV infection, its symptoms, and why early detection and suppression are important. This would be useful given that many patients [25] and primary care providers [26] still lack of knowledge and awareness about very early infection.

In addition to enhanced detection of very early infected adults and adolescents, HIV cure momentum may increase the number of neonates discovered with very early HIV infections. Neonates who acquire HIV infection from their mothers are another key group for HIV cure research efforts as demonstrated by the extended remission of the Mississippi child case [27]. Analogous to adults and adolescents with early infection, this context provides an opportunity in which there is likely a smaller reservoir [28] and may be more amenable to clinical cure research [29]. At the same time, early detection of neonates with newly acquired HIV infection is similarly hampered by a range of problems that include lack of reliable reservoir biomarkers, delays in diagnosis, and insufficient mechanisms to effectively recruit newly infected neonates.

Finally, HIV cure has progressively changed how we talk about HIV in public health messages. Although the resources necessary for implementing HIV control strategies are substantial, available HIV resources have been stable or decreasing in many regions [30, 31]. The field of HIV cure has brought new energy to the HIV field, including scholars, policymakers, and advocates. Qualitative research among HIV-infected individuals has shown that HIV cure research could spark new hope for healing, decreasing barriers to HIV testing and improving retention through the care continuum [32]. One HIV cure community engagement project at the University of North Carolina integrated HIV cure alongside testing, prevention, and other related community HIV campaigns [33]. At the same time, these messages should be cautious and create realistic expectations about the timeframe for development of an HIV cure [34, 35].

## Summary

Given the opportunity presented by a growing HIV cure momentum around the world, we have several recommendations for enabling these policy-program synergies. First, engaging a wide range of global HIV stakeholders, especially in low and middle income settings, will be important for realizing the programmatic synergies articulated above, just as it was in the development of previous interventions [36]. Policymakers make critical decisions about implementation and linkages between research and programs. Second, integrating HIV cure within existing test and treat priorities will help strengthen existing linkages and prevent the development of false dichotomies, such as that which occurred between HIV prevention and treatment in the 1990s [37]. Third, optimizing health systems for very early detection of HIV-infected adults, adolescents, and neonates would be beneficial for both individual clinical and public health outcomes. Finally, the HIV cure global framework requires strengthening multisectoral linkages between clinicians, policymakers, advocates, and other stakeholders in order to develop a comprehensive plan for curing HIV in the future [4].

## Conclusion

HIV cure momentum is building in a range of global settings. While this is a new priority, there are several ways that research towards an HIV cure can strengthen existing HIV policies and practices.

## References

[1] International ASSWGoHIVC, Deeks SG, Autran B, Berkhout B, Benkirane M, Cairns S, Chomont N, Chun TW, Churchill M, Di Mascio M et al. Towards an HIV cure: a global scientific strategy. Nature reviews Immunology. 2012;12(8):607–14. 10.1038/nri3262 PMC3595991 22814509

[2] NIAID Strategic Plan. [http://www.niaid.nih.gov/about/whoweare/planningpriorities/documents/niaidstrategicplan2013.pdf]

[3] Research Toward a Cure Trials June 8, 2015. [http://www.treatmentactiongroup.org/cure/trials]

[4] Tucker JD, Rennie S, Social, Ethical Working Group on HIVC (2014). Social and ethical implications of HIV cure research. Aids.

[5] Global Investment in HIV Cure Research and Development 2014 [http://www.iasociety.org/Web/WebContent/File/Towards_and_HIV_Cure_Tracking_Paper_2014.pdf]

[6] NIH. FY 2014 Trans-NIH Plan for HIV-Related Research. Bethesda: NIH; 2014.

[7] World´s leading HIV/AIDS Scientists and Stakeholders to gather in Washington D.C. to discuss Global Alliance on HIV Cure research [http://www.webcitation.org/6XKxzBPGa]

[8] US-China Program for Research Toward a Cure for HIV/AIDS. Washington, DC. R01 RFA. July 16th, 2014. [http://grants.nih.gov/grants/guide/rfa-files/RFA-AI-14-057.html].

[9] Ananworanich J, Chomont N, Fletcher JLK (2015). Markers of HIV reservoir size and immune activation after treatment in acute HIV infection with and without raltegravir and maraviroc intensification. Journal of Virus Eradication.

[10] International AIDS Society, AVAC, HIV Vaccines & Microbicides Resource Tracking Working Group. Global Investment in HIV Cure Research and Development in 2013: Moving towards greater global investment and collaboration to accelerate research towards a cure for HIV. July 2014.[https://www.iasociety.org/Web/WebContent/File/HIV_Cure_Resource_Tracking_Paper_2013.pdf]

[11] amfAR Announces $100 Million Investment Strategy Aimed at Curing HIV [http://www.webcitation.org/6XKzxZsIv]

[12] UNC-Chapel Hill and GSK announce novel partnership to accelerate search for HIV cure [http://www.webcitation.org/6YVWX1JFU]

[13] Fiebig EW, Wright DJ, Rawal BD, Garrett PE, Schumacher RT, Peddada L, Heldebrant C, Smith R, Conrad A, Kleinman SH et al. Dynamics of HIV viremia and antibody seroconversion in plasma donors: implications for diagnosis and staging of primary HIV infection. Aids. 2003;17(13):1871–9. 10.1097/00002030-200309050-00005 12960819

[14] Ananworanich J, Dube K, Chomont N (2015). How does the timing of antiretroviral therapy initiation in acute infection affect HIV reservoirs?. Current opinion in HIV and AIDS.

[15] Yerly S, Kaiser L, Perneger TV, Cone RW, Opravil M, Chave JP, Furrer H, Hirschel B, Perrin L. Time of initiation of antiretroviral therapy: impact on HIV-1 viraemia. The Swiss HIV Cohort Study. Aids. 2000;14(3):243–9. 10.1097/00002030-200002180-00006 10716500

[16] McNearney T, Hornickova Z, Markham R, Birdwell A, Arens M, Saah A, Ratner L. Relationship of human immunodeficiency virus type 1 sequence heterogeneity to stage of disease. Proceedings of the National Academy of Sciences of the United States of America. 1992;89(21):10247–51. 10.1073/pnas.89.21.10247 PMC50315 1438212

[17] Moir S, Buckner CM, Ho J, Wang W, Chen J, Waldner AJ, Posada JG, Kardava L, O'Shea MA, Kottilil S et al. B cells in early and chronic HIV infection: evidence for preservation of immune function associated with early initiation of antiretroviral therapy. Blood. 2010;116(25):5571–9. 10.1182/blood-2010-05-285528 PMC3031405 20837780

[18] Oxenius A, Price DA, Easterbrook PJ, O'Callaghan CA, Kelleher AD, Whelan JA, Sontag G, Sewell AK, Phillips RE. Early highly active antiretroviral therapy for acute HIV-1 infection preserves immune function of CD8+ and CD4+ T lymphocytes. Proceedings of the National Academy of Sciences of the United States of America. 2000;97(7):3382–7. 10.1073/pnas.97.7.3382 PMC16248 10737796

[19] Deeks SGLS, Havlir DV (2013). The End of AIDS: HIV Infection as a Chronic Disease. Lancet.

[20] Rosenberg NE, Pilcher CD, Busch MP, Cohen MS (2015). How can we better identify early HIV infections?. Current opinion in HIV and AIDS.

[21] Wawer MJGR, Sewankambo NK (2005). Rates of HIV-1 transmission per coital act, by stage of HIV-1 infection, in Rakai, Uganda. Journal of Infectious Disease.

[22] Buzon MJ, Martin-Gayo E, Pereyra F (2014). Long-term antiretroviral treatment initiated in primary HIV-1 infection affects the size, composition and decay kinetics of the reservoir of HIV-1 infected CD4 T cells. J Virol.

[23] Delwart E, Magierowska M, Royz M, Foley B, Peddada L, Smith R, Heldebrant C, Conrad A, Busch M. Homogeneous quasispecies in 16 out of 17 individuals during very early HIV-1 primary infection. Aids. 2002;16(2):189–95. 10.1097/00002030-200201250-00007 11807302

[24] Rieder P, Joos B, von Wyl V, Kuster H, Grube C, Leemann C, Boni J, Yerly S, Klimkait T, Burgisser P et al. HIV-1 transmission after cessation of early antiretroviral therapy among men having sex with men. AIDS. 2010;24(8):1177–83. 10.1097/QAD.0b013e328338e4de 20386427

[25] Remien RH, Higgins JA, Correale J, Bauermeister J, Dubrow R, Bradley M, Steward WT, Seal DW, Sikkema KJ, Kerndt PR et al. Lack of understanding of acute HIV infection among newly-infected persons-implications for prevention and public health: The NIMH Multisite Acute HIV Infection Study: II. AIDS and behavior. 2009;13(6):1046–53. 10.1007/s10461-009-9581-7 PMC2787764 19533323

[26] Kelly JA, Morin SF, Remien RH, Steward WT, Higgins JA, Seal DW, Dubrow R, Atkinson JH, Kerndt PR, Pinkerton SD et al. Lessons learned about behavioral science and acute/early HIV infection. The NIMH Multisite Acute HIV Infection Study: V. AIDS and behavior. 2009;13(6):1068–74. 10.1007/s10461-009-9579-1 PMC2787956 19504179

[27] Persaud D, Gay H, Ziemniak C, Chen YH, Piatak Jr M, Chun TW, Strain M, Richman D, Luzuriaga K. Absence of detectable HIV-1 viremia after treatment cessation in an infant. The New England journal of medicine. 2013;369(19):1828–35. 10.1056/NEJMoa1302976 PMC3954754 24152233

[28] Persaud D, Palumbo PE, Ziemniak C, Hughes MD, Alvero CG, Luzuriaga K, Yogev R, Capparelli EV, Chadwick EG. Dynamics of the resting CD4(+) T-cell latent HIV reservoir in infants initiating HAART less than 6 months of age. Aids. 2012;26(12):1483–90. 10.1097/QAD.0b013e3283553638 PMC3495308 22555165

[29] Rainwater-Lovett K, Luzuriaga K, Persaud D (2015). Very early combination antiretroviral therapy in infants: prospects for cure. Current opinion in HIV and AIDS.

[30] Piot P, Abdool Karim SS, Hecht R, Legido-Quigley H, Buse K, Stover J, Resch S, Ryckman T, Mogedal S, Dybul M et al. Defeating AIDS--advancing global health. Lancet. 2015;386(9989):171–218. 10.1016/S0140-6736(15)60658-4 26117719

[31] Katz I, Glandon D, Wong W, Kargbo B, Ombam R, Singh S, Ramsammy L, Tal-Dia A, Seck I, Osika JS. Lessons learned from stakeholder-driven sustainability analysis of six national HIV programmes. Health Policy Plan. 2014;29(3):379–87. 10.1093/heapol/czt024 23612848

[32] Chu CE, Wu F, He X, Ma Q, Cheng Y, Cai W, Volberding P, Tucker JD. Exploring the social meaning of curing HIV: a qualitative study of people who inject drugs in Guangzhou, China. AIDS research and human retroviruses. 2015;31(1):78–84. 10.1089/aid.2014.0200 PMC4287130 25427547

[33] 2BeatHIV Campaign [www.2BeatHIV.org] Chapel Hill, NC, USA.

[34] Tucker JD, Volberding PA, Margolis DM, Rennie S, Barre-Sinoussi F (2014). Words matter: Discussing research towards an HIV cure in research and clinical contexts. Journal of acquired immune deficiency syndromes.

[35] Rennie S, Siedner M, Tucker JD, Moodley K (2015). The ethics of talking about 'HIV cure'. BMC Med Ethics.

[36] Lo Y, Chu C, Ananworanich J, Excler J, Tucker JD (2015). Stakeholder engagement in HIV cure research: Lessons learned from other HIV interventions and the way forward. AIDS Patient Care & STDs.

[37] MacNeil JM, Anderson S (1998). Beyond the dichotomy: linking HIV prevention with care. Aids.

